# Morphometric study of the T6 vertebra and its three ossification centers in the human fetus

**DOI:** 10.1007/s00276-013-1107-3

**Published:** 2013-03-30

**Authors:** Michał Szpinda, Mariusz Baumgart, Anna Szpinda, Alina Woźniak, Celestyna Mila-Kierzenkowska, Małgorzata Dombek, Adam Kosiński, Marek Grzybiak

**Affiliations:** 1 Department of Normal Anatomy, The Ludwik Rydygier Collegium Medicum in Bydgoszcz, The Nicolaus Copernicus University in Toruń, Karłowicza 24 Street, 85-092 Bydgoszcz, Poland; 2 Department of Medical Biology, The Ludwik Rydygier Collegium Medicum in Bydgoszcz, The Nicolaus Copernicus University in Toruń, Bydgoszcz, Poland; 3 Department of Clinical Anatomy, Medical University in Gdańsk, Gdańsk, Poland

**Keywords:** Typical thoracic vertebra, Ossification center, Dimensions, CT examination, Digital image analysis, Skeletodysplasias, Human fetuses

## Abstract

**Purpose:**

Knowledge on the normative growth of the spine is critical in the prenatal detection of its abnormalities. We aimed to study the size of T6 vertebra in human fetuses with the crown-rump length of 115–265 mm.

**Materials and methods:**

Using the methods of computed tomography (Biograph mCT), digital image analysis (Osirix 3.9) and statistics, the normative growth of the T6 vertebral body and the three ossification centers of T6 vertebra in 55 spontaneously aborted human fetuses (27 males, 28 females) aged 17–30 weeks were studied.

**Results:**

Neither male–female nor right–left significant differences were found. The height, transverse, and sagittal diameters of the T6 vertebral body followed natural logarithmic functions as *y* = −4.972 + 2.732 × ln(age) ± 0.253 (*R* ^2^ = 0.72), *y* = −14.862 + 6.426 × ln(age) ± 0.456 (*R* ^2^ = 0.82), and *y* = −10.990 + 4.982 × ln(age) ± 0.278 (*R* ^2^ = 0.89), respectively. Its cross-sectional area (CSA) rose proportionately as *y* = −19.909 + 1.664 × age ± 2.033 (*R* ^2^ = 0.89), whereas its volumetric growth followed the four-degree polynomial function *y* = 19.158 + 0.0002 × age^4^ ± 7.942 (*R* ^2^ = 0.93). The T6 body ossification center grew logarithmically in both transverse and sagittal diameters as *y* = −14.784 + 6.115 × ln(age) ± 0.458 (*R* ^2^ = 0.81) and *y* = −12.065 + 5.019 × ln(age) ± 0.315 (*R* ^2^ = 0.87), and proportionately in both CSA and volume like *y* = −15.591 + 1.200 × age ± 1.470 (*R* ^2^ = 0.90) and *y* = −22.120 + 1.663 × age ± 1.869 (*R* ^2^ = 0.91), respectively. The ossification center-to-vertebral body volume ratio was gradually decreasing with age. On the right and left, the neural ossification centers revealed the following models: *y* = −15.188 + 6.332 × ln(age) ± 0.629 (*R* ^2^ = 0.72) and *y* = −15.991 + 6.600 × ln(age) ± 0.629 (*R* ^2^ = 0.74) for length, *y* = −6.716 + 2.814 × ln(age) ± 0.362 (*R* ^2^ = 0.61) and *y* = −7.058 + 2.976 × ln(age) ± 0.323 (*R* ^2^ = 0.67) for width, *y* = −5.665 + 0.591 × age ± 1.251 (*R* ^2^ = 0.86) and *y* = −11.281 + 0.853 × age ± 1.653 (*R* ^2^ = 0.78) for CSA, and *y* = −9.279 + 0.849 × age ± 2.302 (*R* ^2^ = 0.65) and *y* = −16.117 + 1.155 × age ± 1.832 (*R* ^2^ = 0.84) for volume, respectively.

**Conclusions:**

Neither sex nor laterality differences are found in the morphometric parameters of evolving T6 vertebra and its three ossification centers. The growth dynamics of the T6 vertebral body follow logarithmically for its height, and both sagittal and transverse diameters, linearly for its CSA, and four-degree polynomially for its volume. The three ossification centers of T6 vertebra increase logarithmically in both transverse and sagittal diameters, and linearly in both CSA and volume. The age-specific reference intervals for evolving T6 vertebra present the normative values of potential relevance in the diagnosis of congenital spinal defects.

## Introduction

The advancement of ultrasound devices allows evaluating most fetal structures, thereby improving the prenatal diagnostics [6–8, 11, 34, 35, 43]. The two methods of computed tomography (CT) and magnetic resonance imaging (MRI) are complementary, in fact often superior, to ultrasonographic scans in assessing suspected spinal anomalies [9, 22, 43]. Accurate knowledge on the normative growth of the spine is critical for diagnosing its congenital defects [13, 15, 24, 39, 46] and skeletodysplasias [38] that produce longitudinal growth imbalance. The height of a typical thoracic vertebra is approximately 3/4 and 4/3 of the heights of the lumbar and cervical vertebrae, respectively [2]. Each vertebra ossifies from the three primary ossification centers, one existing in the vertebral body, and one existing in each neural process [23, 32, 43]. The ossification centers for the neural processes and vertebral bodies develop independently of each other, in a definite topographical progression [2, 43]. Thus, the ossification of vertebral bodies, which commences around the notochord, starts with the thoracolumbar junction in fetuses with the crown-rump length (CRL) of 40–52 mm. From there, the ossification process proceeds in both the cervical and sacral directions [26, 31, 43]. There is a disagreement on the ossification pattern of neural processes, because the following three ossification pathways have been postulated: the first starting simultaneously with the thoracolumbar, cervicothoracic, and superior cervical regions [5, 6]; the second originating in the mid-thoracic region [23], and the third starting with the superior cervical region [3].

Seldom have there been meticulous descriptions in the existing medical literature on morphometric values for thoracic vertebrae in the human fetus [2, 27, 37]. To date, Szpinda et al. [30] have recently published cross-sectional studies concerning the size of vertebral bodies and both body ossification centers [31] and neural ossification centers [32] throughout the fetal spine. Apart from this, these authors performed a precise morphometric study on the three ossification centers of C4 [4] and L3 [33] vertebrae in the human fetus.

Among other thoracic vertebrae, we have specifically looked at the T6 vertebra, being a typical mid-thoracic one. To quantitatively investigate the development of T6 vertebra in fetuses of 115–265 mm CRL, our purposes were set to determine the following:

- age-specific reference intervals for dimensions [height, transverse and sagittal diameters, cross-sectional area (CSA), volume] of its vertebral body,
- age-specific reference intervals for dimensions (transverse and sagittal diameters, CSA, volume) of its three ossification centers,
- the best-fit growth curves for each morphometric parameter studied, and
- the relative growth of the ossification center within the vertebral body (the ossification center-to-vertebral body volume ratio).

## Materials and methods

**Fig. 1:**
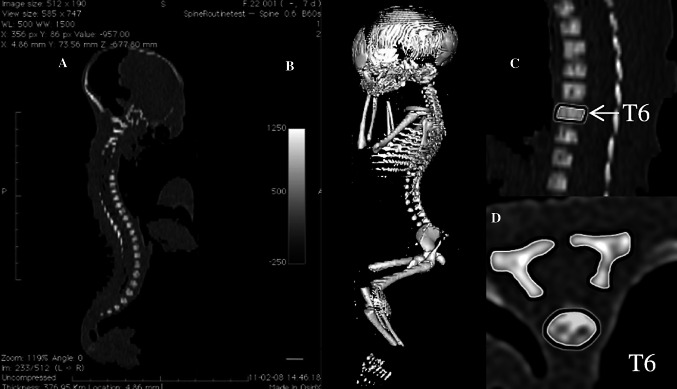
CT of a male fetus aged 23 weeks recorded in DICOM formats (*a*), and assessed by Osirix 3.9 (*b*) in both sagittal (*c*) and horizontal (*d*) planes (*red color* vertebral body, *green color* ossification center of vertebral body, *yellow color* ossification centers of neural processes) (color figure online)

This study encompassed 55 ethnically homogenous human fetuses (27 males, 28 females) of Caucasian racial origin, aged 17–30 weeks (Table 1), which had been derived from spontaneous abortions or stillbirths during the years 1989–2001 because of placental insufficiency. The fetal ages were determined from measurements of the CRL [14], and the known date of the beginning of the last maternal menstrual period. No attempt was done to encourage fetal donation. The use of the fetuses for research was approved by the University Research Ethics Committee (KB 275/2011). On macroscopic examination, both internal and external anatomical malformations, including those related to chromosomal disorders, were ruled out in all included specimens, which were diagnosed as normal. Furthermore, the fetuses studied could not suffer from growth retardation, because the correlation between the gestational age based on the CRL and that calculated by the last menstruation attained the value *R* = 0.98 (*P* < 0.001). After having been immersed in 10 % neutral buffered formalin solution, the fetuses underwent CT examinations with the reconstructed slice width option of 0.4 mm, and 128 slices were acquired simultaneously by Biograph mCT (Siemens). No bones showed an evidence of anomalous development. The CT scans obtained were recorded in DICOM formats (Fig. 1a), allowing us to create both three-dimensional reconstructions and the morphometric analysis of chosen objects. The gray scale of obtained CT images in Hounsfield units varied from −275 to −134 for a minimum, and from +1,165 to +1,558 for a maximum.

**Table 1:** Distribution of the fetuses studied

Gestational age^a^ (weeks)	Crown-rump length (CRL) (mm)				Number	Sex	
	Mean	SD	Min	Max		Male	Female
17	115.00		115.00	115.00	1	0	1
18	133.33	5.77	130.00	140.00	3	1	2
19	149.50	3.82	143.00	154.00	8	3	5
20	161.00	2.71	159.00	165.00	4	2	2
21	174.75	2.87	171.00	178.00	4	3	1
22	185.00	1.41	183.00	186.00	4	1	3
23	197.60	2.61	195.00	202.00	5	2	3
24	208.67	3.81	204.00	213.00	9	5	4
25	214.00		214.00	214.00	1	0	1
26	229.00	5.66	225.00	233.00	2	1	1
27	239.17	3.75	235.00	241.00	6	6	0
28	249.50	0.71	249.00	250.00	2	0	2
29	253.00	0.00	253.00	253.00	2	0	2
30	263.25	1.26	262.00	265.00	4	3	1
Total					55	27	28

^a^The gestational age based on the CRL and that calculated by known date of the beginning of the last maternal menstrual period was highly correlated (*R* = 0.98; *P* < 0.001)

**Fig. 2:**
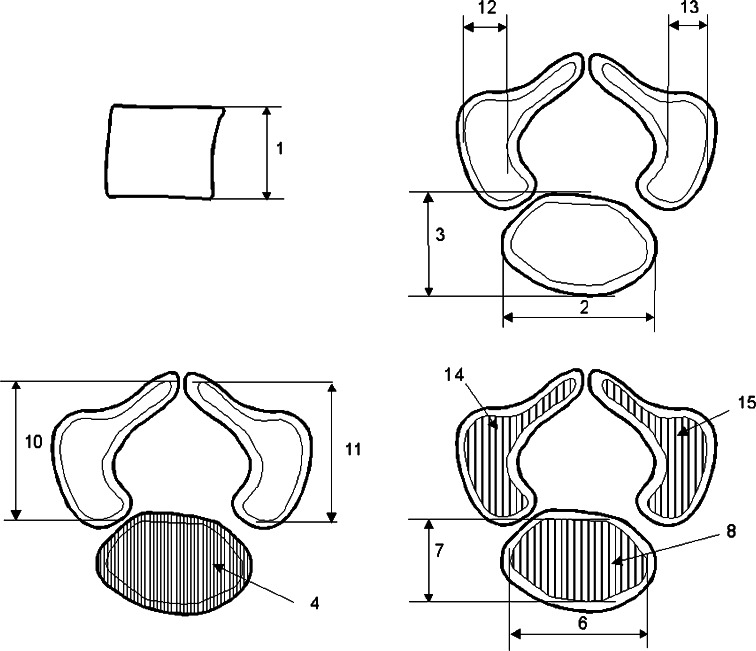
Diagram showing different measurements (apart from volumes) of the T6 vertebral body and the three ossification centers (the numbers according to the definitions of measurements in the text): height (*1*), transverse (*2*) and sagittal (*3*) diameters, and CSA (*4*) of the T6 vertebral body; transverse (*6*) and sagittal (*7*) diameters, and CSA (*8*) of the T6 body ossification center; lengths (*10*, *11*), widths (*12*, *13*) and CSAs (*14*, *15*) of the right and left neural ossification centers, respectively

As a result, the window width (WW) ranged from 1,404 to 1,692, and the window level (WL) varied from +463 to +712. Measurements of the spine could be obtained only after identifying T6 vertebra. Next, DICOM formats were evaluated using digital image analysis of Osirix 3.9 (Fig. 1b) with estimating linear (sagittal and transverse diameters, height, length, and width), two-dimensional (CSA), and three-dimensional (volume) parameters of T6 vertebra (Fig. 1c, d). The contouring procedure for each T6 vertebral body and the three ossification centers were outlined with a cursor and stored. The diagram (Fig. 2) presents different measurements (apart from volumes) of the T6 vertebral body and the three ossification centers of T6 vertebra.

The following five parameters of the T6 vertebral body (Fig. 2) for each fetus were assessed:

1. height (in mm), corresponding to the distance between the superior and inferior borderlines of the vertebral body (in sagittal projection),
2. transverse diameter (in mm), corresponding to the distance between the left and right borderlines of the vertebral body (in transverse projection),
3. sagittal diameter (in mm), corresponding to the distance between the anterior and posterior borderlines of the vertebral body (in sagittal projection),
4. cross-sectional area (in mm^2^), traced around the vertebral body (in transverse projection), and
5. volume (in mm^3^).
  In addition, the following 12 parameters of the 3 ossifications centers were assessed for each individual:
    within the vertebral body (6–9):
6. transverse diameter (in mm), corresponding to the distance between the left and right borderlines of the ossification center (in transverse projection),
7. sagittal diameter (in mm), corresponding to the distance between the anterior and posterior borderlines of the ossification center (in sagittal projection),
8. cross-sectional area (in mm^2^), traced around the ossification center (in transverse projection),
9. volume (in mm^3^), and
  within the right and left neural processes (10–17):
10. right and left lengths (in mm), corresponding to the distance between the anterior and posterior borderlines of the ossification center (in transverse projection),
11. right and left widths (in mm), corresponding to the distance between the left and right borderlines of the ossification center (in transverse projection),
12. right and left CSAs (in mm^2^), traced around the ossification center (in transverse projection),
13. right and left volumes (in mm^3^).

Since both volumes and CSAs did not represent derived parameters, the present study provides only direct measurements, instead of deduced, extrapolated data obtained through a series of indirect measurements. In a continuous effort to minimize measurements and observer bias, all measurements were done by one researcher (M.B.). Each measurement was repeated three times under the same conditions, but at different times, and then averaged. The differences between repeated measurements, as the intra-observer variation, were evaluated by the one-way ANOVA test for paired data. The results obtained were subjected to statistical analysis. All the parameters studied were plotted against gestational age, to construct their growth dynamics. The relative growth, both the T6 vertebral body and its ossification center was expressed as the sagittal-to-transverse diameter ratios and the ossification center-to-vertebral body volume ratio. The data obtained were checked for normality of distribution using the Kolmogorov–Smirnov test and homogeneity of variance with the use of Levene’s test. As a consequence of the statistical analysis, Student’s *t* test was used to examine the influence of sex on the values obtained. To examine sex differences, we checked the possible differences between the following five age groups: 17–19, 20–22, 23–25, 26–28, and 29–30 weeks. Next, we tested sex differences for the whole examined group, without taking into account the fetal ages. To check whether significant differences existed with age, the one-way ANOVA test for unpaired data, and then post hoc RIR Tukey comparisons were used for the five age groups. Linear and nonlinear regression analysis was used to derive the best-fit curve (*y*) for each parameter against gestational age (*x*), with estimating coefficients of determination (*R* ^2^) between each parameter and fetal age. Different regressions were computed for every parameter growth, but the best ones proved to be as follows: natural logarithmic functions for all linear measurements (height, transverse and sagittal diameters of vertebral body; transverse and sagittal diameters of body ossification center; length and width of neural ossification centers), linear functions for both two-dimensional measurements (CSAs of vertebral body, body ossification center and neural ossification centers) and volumes of the three ossification centers, and a four-degree polynomial function for the vertebral body volume. Since all the examined linear parameters were characterized by a gradually decreasing growth rate, natural logarithmic functions expressed as *y* = ln(*x*) or *y* = log_e_(*x*) (e constant in log_e_ as Euler’s number approximately equals 2.71828183) were much better than possible square root models or quadratic functions with a negative coefficient of power 2. It should be noticed that the natural logarithmic function *y* = ln(*x*) is the inverse function of the exponential function *y* = e^*x*^. This means that *y* = ln(*x*) is equivalent to *x* = e^*y*^. From a mathematical point of view, the growth dynamics typical of the natural logarithm of fetal age are one-to-one (for each *y* there is one and only one *x*), continuous, and increasing. Apart from this, they indicate a declining rate of change, being expressed by a concave down graph, with age more and more deviating from the straight line *y* = *x*. On the contrary, in a linear function the rate of growth remains the same across the graph, while in a four-degree polynomial function the rate of growth gradually increases with age. Differences were considered significant at *P* < 0.05.

## Results

**Fig. 3:**
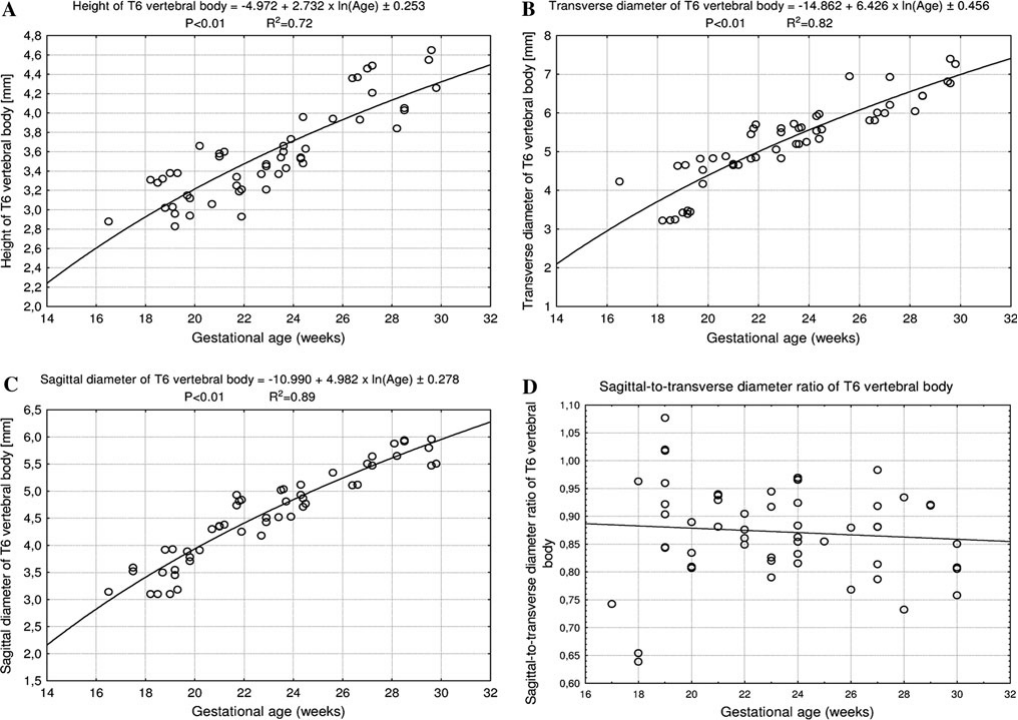
Regression lines for height (**a**), transverse (**b**) and sagittal (**c**) diameters, and sagittal-to-transverse diameter ratio (**d**) of the T6 vertebral body

**Fig. 4:**
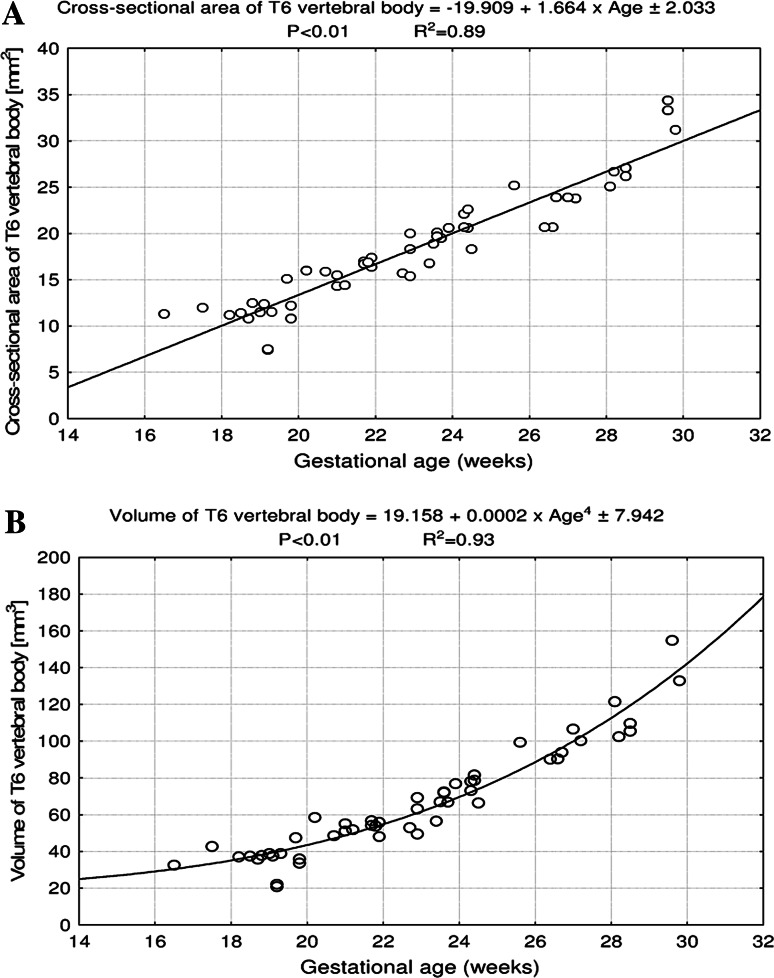
Regression lines for CSA (**a**) and volume (**b**) of the T6 vertebral body

No statistically significant differences were found in evaluating intra-observer reproducibility of the spinal measurements (*P* > 0.05, the one-way ANOVA test for paired data and post hoc RIR Tukey test). In addition, no significant difference was observed in the values of the parameters studied according to sex (*P* > 0.05, Student’s *t* test), so the morphometric values for the T6 vertebral body (Table 2) and the three ossification centers of T6 vertebra (Tables 3, 4) have been summarized for both sexes. By contrast, advancing gestational age was characterized by a statistically significant increase (*P* < 0.01, the one-way ANOVA test for unpaired data and post hoc RIR Tukey test) in values of all measurements. The numerical data correlated to age showed growth dynamics, presented by specific best-fit growth curves (Figs. 3, 4, 6, 7, 8, 9).

**Table 2:** Morphometric parameters of the T6 vertebral body

Age (weeks)	*n*	Height (mm)		Transverse diameter (mm)		Sagittal diameter (mm)		CSA (mm^2^)		Volume (mm^3^)	
		Mean	SD	Mean	SD	Mean	SD	Mean	SD	Mean	SD
17	1	2.88		4.23		3.84		11.30		32.54	
18	3	3.48	0.14	4.74	1.32	4.34	1.08	13.07	2.57	45.60	10.35
19	8	3.15	0.21	3.69	0.60	3.84	1.14	10.63	2.04	33.69	7.55
		↓ (*P* < 0.05)		↓ (*P* < 0.01)		↓ (*P* < 0.05)		↓ (*P* < 0.001)		↓ (*P* < 0.001)	
20	4	3.22	0.31	4.59	0.31	3.82	0.09	13.53	2.43	43.92	11.50
21	4	3.45	0.26	4.72	0.11	4.35	0.03	15.03	0.80	51.68	2.62
22	4	3.17	0.17	5.40	0.38	4.71	0.31	16.93	0.41	53.65	3.92
		↓ (*P* < 0.05)		↓ (*P* < 0.001)		↓ (*P* < 0.01)		↓ (*P* < 0.001)		↓ (*P* < 0.01)	
23	5	3.37	0.10	5.34	0.38	4.59	0.42	17.24	1.92	58.30	8.02
24	9	3.61	0.16	5.52	0.30	4.94	0.26	20.53	1.19	74.07	5.16
25	1	3.63		5.58		4.77		18.30		66.43	
		↓ (*P* < 0.01)		↓ (*P* < 0.001)		↓ (*P* < 0.001)		↓ (*P* < 0.001)		↓ (*P* < 0.01)	
26	2	4.15	0.30	6.38	0.81	5.73	0.87	22.95	3.18	94.77	6.39
27	6	4.12	0.47	5.96	0.68	5.20	0.40	23.90	5.87	99.98	32.33
28	2	4.84	1.41	7.04	1.40	5.77	0.16	25.90	1.13	112.01	13.40
		↓ (*P* < 0.05)		↓ (*P* < 0.05)		↓ (*P* < 0.01)		↓ (*P* < 0.001)		↓ (*P* < 0.01)	
29	2	4.04	0.01	6.45	0.01	5.93	0.01	26.65	0.64	107.67	2.95
30	4	4.64	0.34	7.07	0.32	5.94	0.70	34.08	2.58	158.17	18.87

**Table 3:** Morphometric parameters of the T6 body ossification center

Age (weeks)	*n*	T6 body ossification center							
		Transverse diameter (mm)		Sagittal diameter (mm)		Cross-sectional area (mm^2^)		Volume (mm^3^)	
		Mean	SD	Mean	SD	Mean	SD	Mean	SD
17	1	3.24		2.96		6.90		7.72	
18	3	3.82	1.13	3.55	1.20	7.67	1.96	11.09	2.61
19	8	2.93	0.47	2.51	0.32	6.82	1.30	8.83	1.07
		↓ (*P* < 0.01)		↓ (*P* < 0.01)		↓ (*P* < 0.001)		↓ (*P* < 0.01)	
20	4	3.95	0.14	3.05	0.28	8.23	0.39	11.00	0.88
21	4	3.41	0.02	3.16	0.23	9.23	0.36	11.58	0.73
22	4	4.56	0.50	3.73	0.24	11.53	1.25	15.08	2.30
		↓ (*P* < 0.01)		↓ (*P* < 0.01)		↓ (*P* < 0.01)		↓ (*P* < 0.001)	
23	5	4.46	0.30	3.78	0.45	11.28	2.12	15.20	2.74
24	9	4.49	0.31	4.02	0.26	12.81	1.37	18.26	2.52
25	1	4.64		3.87		12.70		17.60	
		↓ (*P* < 0.001)		↓ (*P* < 0.001)		↓ (*P* < 0.001)		↓ (*P* < 0.001)	
26	2	5.49	0.51	4.70	0.92	15.90	2.40	22.55	2.90
27	6	5.20	0.58	4.29	0.58	16.08	1.97	21.87	2.72
28	2	5.91	1.19	5.22	0.81	17.95	0.78	25.55	0.49
		↓ (*P* < 0.01)		↓ (*P* < 0.01)		↓ (*P* < 0.001)		↓ (*P* < 0.001)	
29	2	5.78	0.00	4.87	0.01	20.85	0.49	26.85	0.21
30	4	5.98	0.60	4.67	0.49	20.23	1.38	25.73	1.82

**Table 4:** Morphometric parameters of the neural ossification centers of T6 vertebra

Age (weeks)	*n*	Right neural ossification center								Left neural ossification center							
		Length (mm)		Width (mm)		Cross-sectional area (mm^2^)		Volume (mm^3^)		Length (mm)		Width (mm)		Cross-sectional area (mm^2^)		Volume (mm^3^)	
		Mean	SD	Mean	SD	Mean	SD	Mean	SD	Mean	SD	Mean	SD	Mean	SD	Mean	SD
17	1	2.98		1.31		4.40		5.55		3.19		1.42		4.00		5.73	
18	3	3.69	0.81	1.41	0.08	5.87	0.49	9.26	1.72	3.48	0.80	1.42	0.06	4.73	1.07	6.79	1.84
19	8	3.03	0.31	1.39	0.06	5.08	0.76	6.65	0.98	2.95	0.55	1.59	0.07	4.22	1.38	5.24	0.94
		↓		↓		↓		↓		↓		↓		↓		↓	
		(*P* < 0.001)	0.53	(*P* < 0.01)	0.28	(*P* < 0.01)	1.17	(*P* < 0.01)	0.87	(*P* < 0.001)	0.22	(*P* < 0.01)	0.14	(*P* < 0.001)	0.70	(*P* < 0.01)	0.35
20	4	3.84		1.79		5.68		6.79		4.03		2.03		7.03		6.96	
21	4	4.25	0.20	1.65	0.04	6.58	0.46	6.62	0.11	4.58	0.50	1.92	0.05	5.53	0.75	5.73	0.26
22	4	4.27	0.70	2.10	0.24	6.65	1.37	7.96	1.72	4.41	0.36	2.19	0.04	5.88	2.19	8.29	1.13
		↓		↓		↓		↓		↓		↓		↓		↓	
		(*P* < 0.01)		(*P* < 0.01)		(*P* < 0.001)		(*P* < 0.001)		(*P* < 0.05)		(*P* < 0.05)		(*P* < 0.001)		(*P* < 0.001)	
23	5	4.45	0.41	2.30	0.69	8.16	1.32	10.18	2.91	4.49	0.63	2.50	0.58	8.66	1.78	10.31	2.41
24	9	4.99	0.90	2.29	0.50	9.22	1.99	11.34	1.74	4.79	0.60	2.49	0.37	10.10	1.59	12.42	1.92
25	1	4.80		2.72		9.60		10.70		5.30		2.71		10.10		10.20	
		↓		↓		↓		↓		↓		↓		↓		↓	
		(*P* < 0.001)		(*P* < 0.01)		(*P* < 0.001)		(*P* < 0.001)		(*P* < 0.001)		(*P* < 0.05)		(*P* < 0.01)		(*P* < 0.001)	
26	2	6.08	0.59	2.78	0.47	14.05	3.04	18.65	2.19	5.92	0.66	2.81	0.45	12.85	1.63	16.80	0.85
27	6	5.30	0.85	2.45	0.49	9.73	1.91	13.02	3.45	5.54	1.23	2.56	0.77	10.53	2.77	13.97	3.20
28	2	6.53	0.67	2.52	0.56	14.40	2.69	16.65	3.32	6.28	1.04	2.78	0.45	11.55	3.32	15.60	0.99
		↓		↓		↓		↓		↓		↓		↓		↓	
		(*P* < 0.05)		(*P* < 0.01)		(*P* < 0.01)		(*P* < 0.01)		(*P* < 0.01)		(*P* < 0.05)		(*P* < 0.05)		(*P* < 0.01)	
29	2	5.94	0.01	2.59	0.01	10.30	0.14	11.95	0.21	6.19	0.01	2.57	0.01	11.90	0.14	19.35	0.07
30	4	6.12	0.83	2.54	0.41	11.43	1.84	16.40	3.73	6.22	0.79	2.48	0.49	12.10	1.89	15.70	2.93

The size of the T6 vertebral body has been presented in Table 2. The values of the T6 vertebral body height grew from 2.88 to 4.64 ± 0.34 mm for fetuses aged 17 and 30 weeks, respectively. With advancing gestational age, an increase in height (Fig. 3a) followed logarithmically as *y* = −4.972 + 2.732 × ln(age) ± 0.253 (*R* ^2^ = 0.72). Between ages of 17 and 30 weeks, the transverse diameter of the T6 vertebral body (Fig. 3b) increased from 4.23 to 7.0 ± 0.32 mm, according to the natural logarithmic function: *y* = −14.862 + 6.426 × ln(age) ± 0.456 (*R* ^2^ = 0.82). During the study period, the values of sagittal diameter of the T6 vertebral body (Fig. 3c) grew logarithmically from 3.84 to 5.94 ± 0.70 mm, in accordance with the formula: *y* = −10.990 + 4.982 × ln(age) ± 0.278 (*R* ^2^ = 0.89). As a result, at ages of 17 and 30 weeks, the growth velocities (mm per week) for height, transverse and sagittal diameters of the T6 vertebral body gradually decreased with advancing fetal age (*P* < 0.01, the one-way ANOVA test for unpaired data and post hoc RIR Tukey test), from 0.16 to 0.09 mm, from 0.37 to 0.22 mm, and from 0.28 to 0.17 mm, respectively. The relative growth of the T6 vertebral body did not turn out to be proportionate because its transverse diameter grew much faster than its sagittal diameter. This was expressed by the decrement of the sagittal-to-transverse diameter ratio (Fig. 3d) from 0.88 ± 0.12 to 0.86 ± 0.11 (*P* < 0.05, the one-way ANOVA test for unpaired data and post hoc RIR Tukey test).

The values of CSA of the T6 vertebral body (Fig. 4a) varied from 11.30 mm^2^ in a fetus aged 17 weeks to 34.08 ± 2.58 mm^2^ in fetuses aged 30 weeks, and modeled the linear function *y* = −19.909 + 1.664 × age ± 2.033 (*R* ^2^ = 0.89). At the same time, the volumetric growth of the T6 vertebral body (Fig. 4b), from 32.54 to 158.17 ± 18.87 mm^3^, followed the four-degree polynomial regression *y* = 19.158 + 0.0002 × age^4^ ± 7.942 (*R* ^2^ = 0.93).

**Fig. 5:**
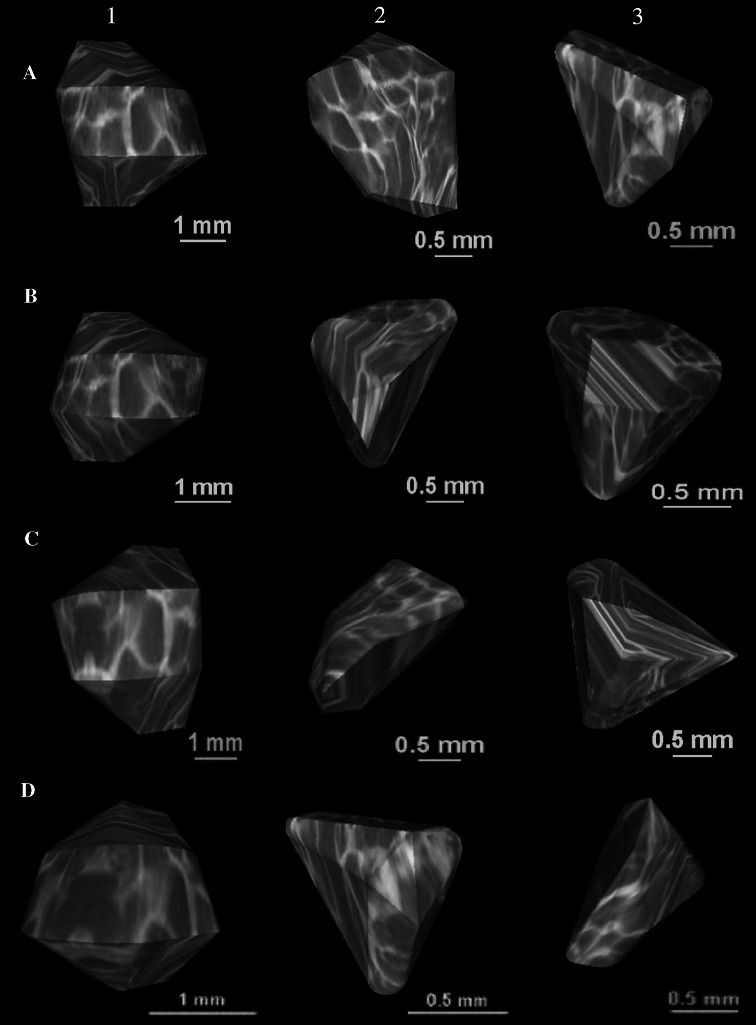
Ossification centers of the vertebral body (*1*), and right (*2*) and left (*3*) neural processes of T6 vertebra in fetuses aged 17 weeks (*a*), 22 weeks (*b*), 26 weeks (*c*), and 30 weeks (*d*) (color figure online)

**Fig. 6:**
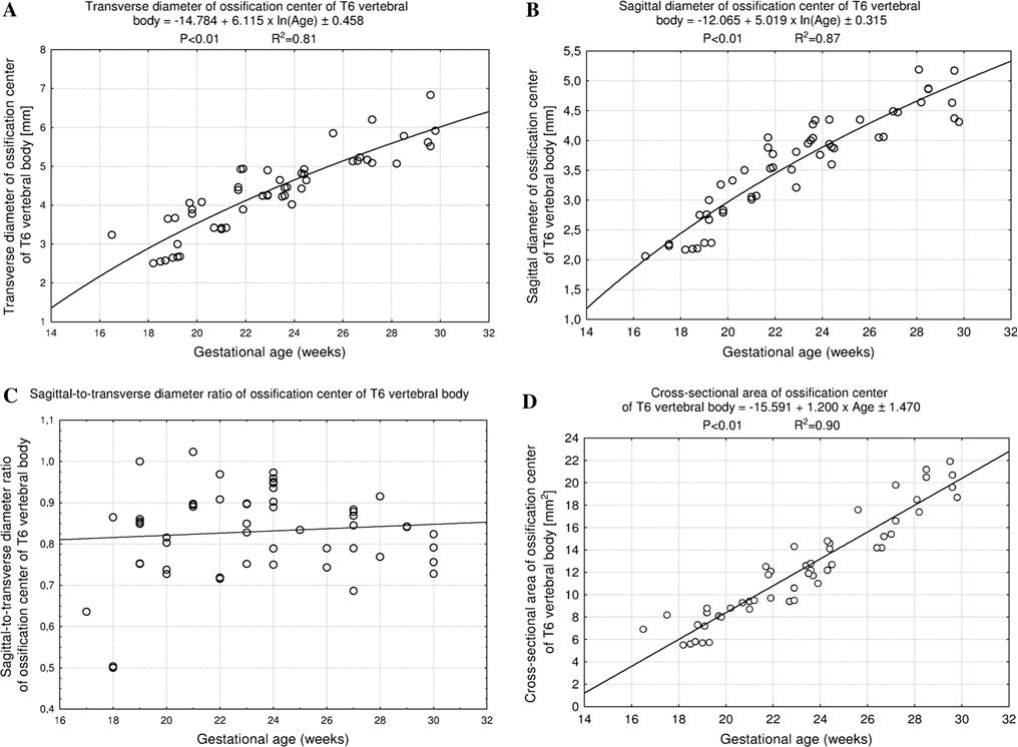
Regression lines for transverse (**a**) and sagittal (**b**) diameters, sagittal-to-transverse diameter ratio (**c**), and CSA (**d**) of the T6 body ossification center

The numerical data of the ossification center of the T6 vertebral body have been presented in Table 3, while Fig. 5 presents the three ossification centers of T6 vertebra within its body (1), and right (2) and left (3) neural processes in fetuses aged 17, 22, 26, and 30 weeks, respectively. During the analyzed period, the transverse (Fig. 6a) and sagittal (Fig. 6b) diameters of the ossification center of the T6 vertebral body increased logarithmically, from 3.24 to 5.98 ± 0.60 mm, and from 2.96 to 4.67 ± 0.49 mm, in accordance with the following models: *y* = −14.784 + 6.115 × ln(age) ± 0.458 (*R* ^2^ = 0.81) and *y* = −12.065 + 5.019 × ln(age) ± 0.315 (*R* ^2^ = 0.87), respectively. As a result, the growth dynamics for both transverse and sagittal diameters declined with gestational age, from 0.35 to 0.21 mm/week, and from 0.29 to 0.17 mm/week (*P* < 0.01, the one-way ANOVA test for unpaired data and post hoc RIR Tukey test), respectively. During the study period, the sagittal-to-transverse diameter ratio of the body ossification center (Fig. 6c) increased from 0.81 ± 0.07 to 0.85 ± 0.08 (*P* < 0.05, the one-way ANOVA test for unpaired data and post hoc RIR Tukey test).

**Fig. 7:**
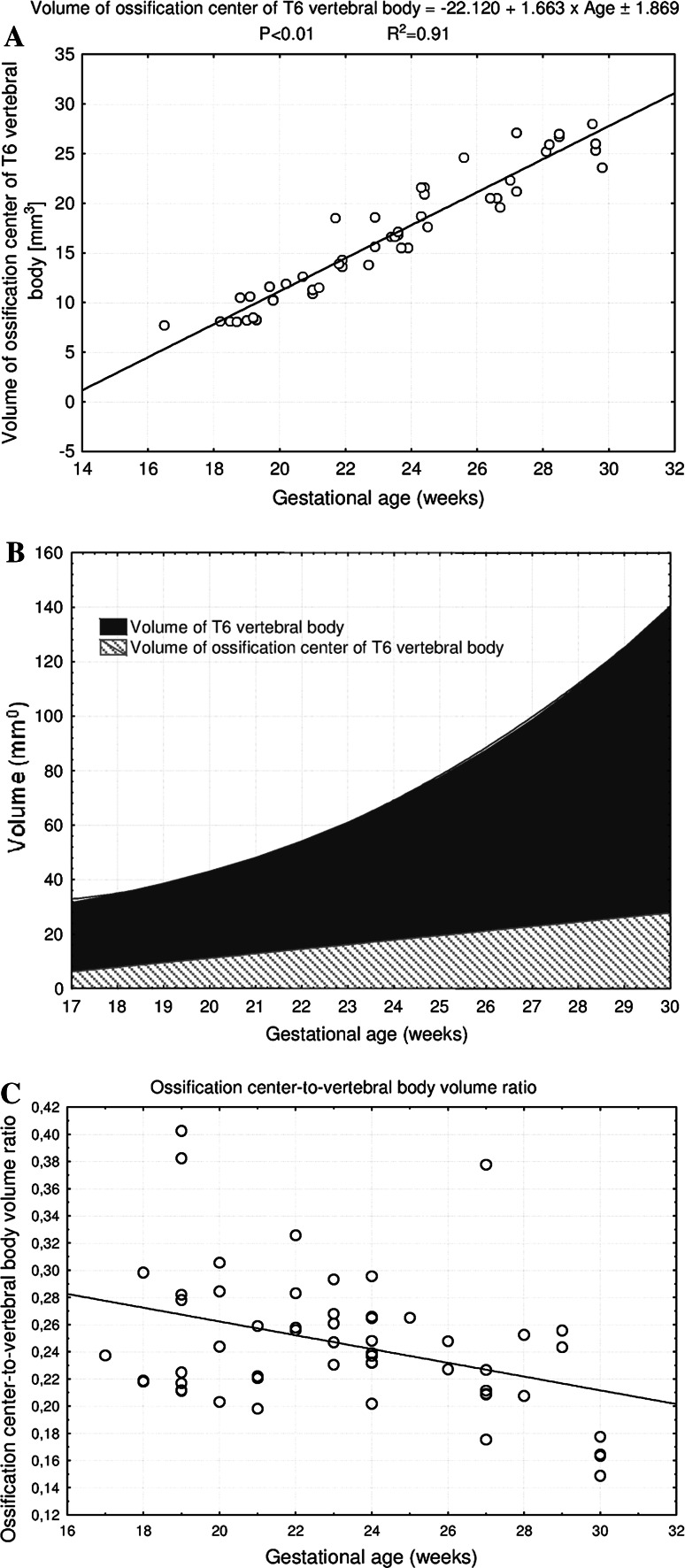
Regression lines for volume of the ossification center of the T6 vertebral body (**a**), when compared to the T6 vertebral body volume (**b**), and the ossification center-to-vertebral body volume ratio (**c**) (color figure online)

The CSA of the ossification center of T6 vertebral body (Fig. 6d) increased proportionately from 6.90 mm^2^ in a fetus aged 17 weeks to 20.23 ± 1.38 mm^2^ in fetuses aged 30 weeks, according to the linear model *y* = −15.591 + 1.200 × age ± 1.470 (*R* ^2^ = 0.90). The volumetric growth of the ossification center (Fig. 7a), from 7.72 to 25.73 ± 1.82 mm^3^, followed linearly as *y* = −22.120 + 1.663 × age ± 1.869 (*R* ^2^ = 0.91).

The volumetric growth of the T6 vertebral body and its ossification center (Fig. 7b) was expressed in a relative fashion by the ossification center-to-vertebral body volume ratio. As plotted in Fig. 7c, its value gradually decreased from 0.28 ± 0.07 to 0.21 ± 0.05 during the study period (*P* < 0.01, the one-way ANOVA test for unpaired data and post hoc RIR Tukey test).

**Fig. 8:**
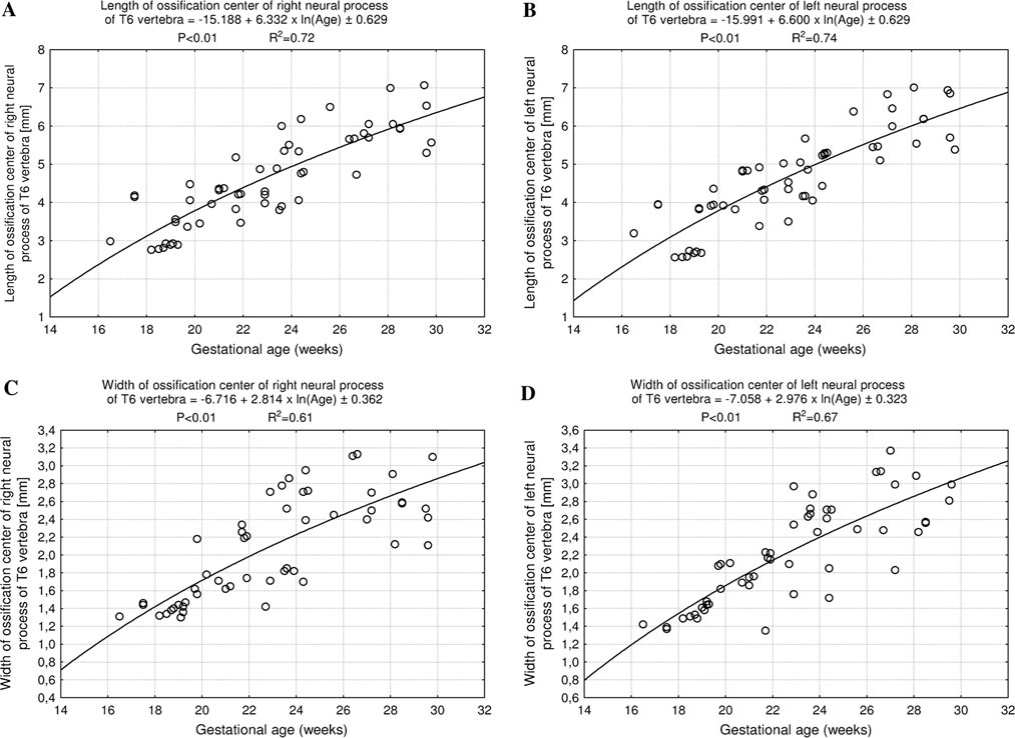
Regression lines for length on the* right* (**a**) and* left* (**b**), and for width on the* right* (**c**) and* left* (**d**) of the neural ossification centers

**Fig. 9:**
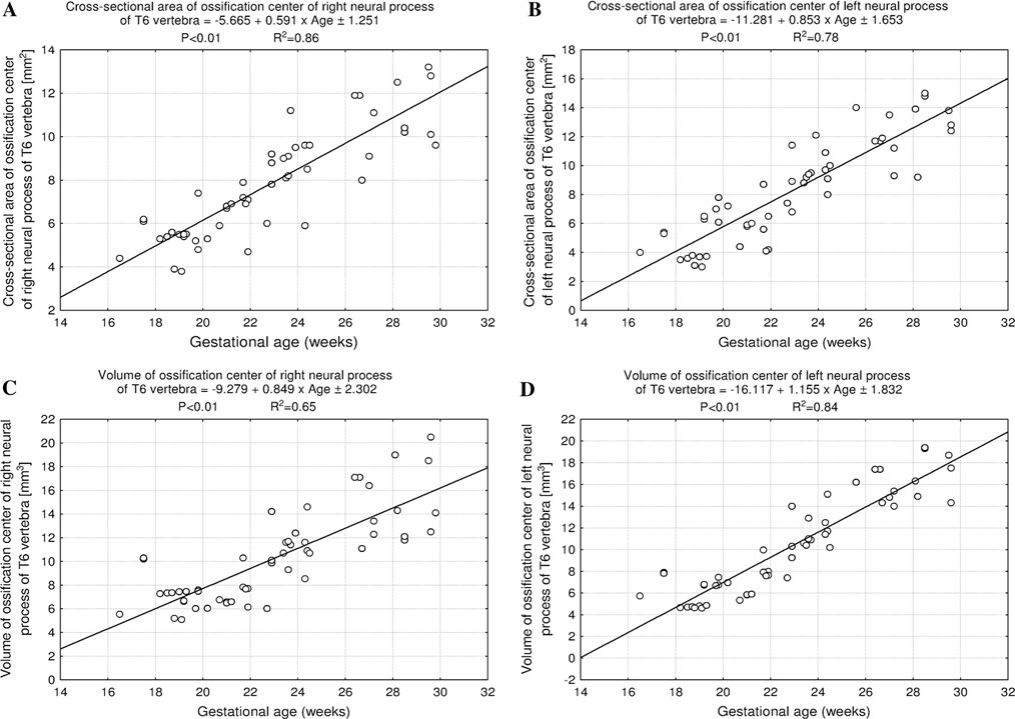
Regression lines for CSA on the* right* (**a**) and* left* (**b**), and for volume on the* right* (**c**) and* left* (**d**) of the neural ossification centers

The size of the neural ossification centers has been given in Table 4. Although the right–left differences for the whole group were not statistically significant, the findings have been presented separately for each neural process, because of their great inter-individual variability (Table 4). The neural ossification center increased in length from 2.98 to 6.12 ± 0.83 mm on the right (Fig. 8a), and from 3.19 to 6.22 ± 0.79 mm on the left (Fig. 8b), in accordance with the natural logarithmic functions: *y* = −15.188 + 6.332 × ln(age) ± 0.629 (*R* ^2^ = 0.72) and *y* = −15.991 + 6.600 × ln(age) ± 0.629 (*R* ^2^ = 0.74), respectively. Its width grew from 1.31 to 2.54 ± 0.41 mm on the right (Fig. 8c), and from 1.42 to 2.48 ± 0.49 mm on the left (Fig. 8d), following the natural logarithmic functions: *y* = −6.716 + 2.814 × ln(age) ± 0.362 (*R* ^2^ = 0.61) and *y* = −7.058 + 2.976 × ln(age) ± 0.323 (*R* ^2^ = 0.67), respectively. The CSA of the neural ossification center showed an increase from 4.40 to 11.43 ± 1.84 mm^2^ on the right (Fig. 9a), and from 4.00 to 12.10 ± 1.89 mm^2^ on the left (Fig. 9b), in correspondence with the linear functions: *y* = −5.665 + 0.591 × age ± 1.251 (*R* ^2^ = 0.86) and *y* = −11.281 + 0.853 × age ± 1.653 (*R* ^2^ = 0.78), respectively. The volumetric growth of the right (Fig. 9c) and left (Fig. 9d) neural ossification centers ranged from 5.55 to 16.40 ± 3.73 mm^3^, and from 5.73 to 15.70 ± 2.93 mm^3^ respectively, following the linear functions *y* = −9.279 + 0.849 × age ± 2.302 (*R* ^2^ = 0.65), and *y* = −16.117 + 1.155 × age ± 1.832 (*R* ^2^ = 0.84).

## Discussion

This study has presented a cross-sectional interpretation of the longitudinal growth of 17 examined parameters of the T6 vertebra based on the evidence from 55 fetuses at ages of 17–30 weeks. As a result, it is not a true representation of growth in itself, but a populational perspective. The main limitation of the present study is a relatively narrow fetal age, ranging from 17 to 30 weeks of gestation. Were we able to collect a larger fetal sample size with a wider age range, it would be possible to improve the growth curves obtained. Another partial limitation is that all measurements were conducted by a single observer in a blind fashion.

The vertebral bodies originate from an axial cartilaginous skeleton that encloses the notochord. The notochord, also called chordamesoderm, develops from epiblast cells of the medial part of the primitive node during the third week of gestation [29]. As early as in the stage 8 embryo, the notochordal cells align themselves in the midline along the rostral–caudal axis to form a notochordal plate, which will subsequently fold to create the notochord with its central canal [17]. The notochord induces formation of the spine, by participating in both the process of chondrogenesis of vertebral bodies and the formation of the nucleus pulposus. Apart from this, the early notochord is critical for the maintenance and development of the neural floor plate and the induction of motor neurons [29]. In the 5-week human embryo, the notochord displays a solid rod of cells extending throughout the developing spine. The replacement of notochordal tissue by surrounding cartilage gradually proceeds between the 7th and 12th week of prenatal life, resulting in the growth and coalescence of two chondrification centers into one body ossification center [25]. Although the notochord disappears completely on the turn of the first trimester [3], the earliest body ossification center is already formed in the 8-week fetus, around the remnants of the notochord. Afterwards, at the beginning of the second trimester, the body ossification center progresses both centrifugally to increase its size and centripetally to invade the formerly avascular notochordal region [28].

Since the spine starts to mineralize in the eighth week of pregnancy [1, 5], it can be visualized by ultrasound from the ninth week. During the 11th week of gestation, a fetus presents ossification centers within both the T2–L2 vertebral bodies and the C1–L1 neural processes [3]. The ossification timing observed by embryologists and sonographers was different, because histological studies showed mineralization in much younger specimens [5, 16]. According to Vignolo et al. [40], the ossification timing was significantly earlier in females than in males. With relation to S5 vertebra, its body ossification center and neural ossification centers were visualized in 42.9 % and 28.6 % of the female fetuses respectively, while in no one male fetus at the same gestational age. In this regard, our findings do not correspond with the medical literature, since the statistically insignificant differences in sex were found in the material under examination. In our opinion, the possible explanation to this may be partly attributed both to the great inter-individual variability of the fetuses studied and to the different methods used.

Evaluation of the fetal spine in both transverse and parasagittal planes constitutes an integral part of routine ultrasound scanning [26]. Growth dynamics for the thoracic spine length have previously been reported to be linear [19], quadratic [2], or exponential [26], when correlated with advancing gestational ages. As reported by Tulsi [37], between 2–4 and 17–19 years, the heights of all thoracic vertebrae continued to increase by 36–47 %. Bagnall et al. [2] showed that in fetuses aged 8–26 weeks, the thoracic spine length grew from 25 to 60 mm, being precisely expressed by the quadratic function *y* = −28.07 + 247.67 × age − 691.97 × age^2^ (*R* = 0.99, age in years) with a negative coefficient of power 2, indicating a gradually decreasing growth rate. Even though the whole presacral spine slowed down in its development, the thoracic part still slowed down to approximately twice the growth rate in both the lumbar and cervical parts [2]. Therefore, in fetuses at the age of 8 and 26 weeks, the thoracic part of the spine was, respectively, 2.5 and two times longer than its lumbar part. Furthermore, the length of the “average” thoracic unit (vertebra plus disc) at 26 weeks of gestation reached the value of 5.0 mm.

In the present study, the height, and both transverse and sagittal diameters of the T6 vertebral body did not generate linear, quadratic or exponential functions on the nomograms. In fact, the best-fit growth models of the T6 vertebral body were the following natural logarithmic functions: *y* = −4.972 + 2.732 × ln(age) ± 0.253 for its height, *y* = −14.862 + 6.426 × ln(age) ± 0.456 for its transverse diameter, and *y* = −10.990 + 4.982 × ln(age) ± 0.278 for its sagittal diameter. As a consequence, their growth velocities were gradually declining with age, as previously reported by Bagnall et al. [2].

In the material under examination, the T6 vertebral body did not show a proportionate evolution because the sagittal-to-transverse diameter ratio decreased from 0.88 ± 0.12 to 0.86 ± 0.11 during the analyzed period. Since both the transverse and sagittal diameters of the T6 vertebral body increased logarithmically, its CSA being approximately a product of these two diameters, generated the linear fashion *y* = −19.909 + 1.664 × age ± 2.033. As with relation to the T6 vertebral body, the C4 and L3 ones were found to increase logarithmically in height and both sagittal and transverse diameters, and linearly in CSA [4, 33].

The overall rate of growth of vertebral bodies was best determined by measuring their volume [37]. Schild et al. [27] presented a three-dimensional sonographic volume calculation of the T12 vertebral body in fetuses aged 16–37 weeks. Its growth in volume varied from 0.047 to 2.311 ml, in correspondence (*P* < 0.01) with the exponential function *y* = exp (2.785 − 86.94/age) (*R* ^2^ = 0.918). Interestingly enough, in the material under examination, the T6 vertebral body volume varied from 32.54 to 158.17 ± 18.87 mm^3^, with the model of choice for volume expressed as the four-degree polynomial function *y* = 19.158 + 0.0002 × age^4^ ± 7.942. In our opinion, this model may probably result from multiplying the three values for height, transverse and sagittal diameters, each changing logarithmically. Of note, the volumetric growth of the C4 vertebral body followed a four-degree polynomial function [4], whereas that of the L3 vertebral body varied two-degree polynomially [33]. Postnatally, an increase in volume of the thoracic vertebrae by 76 % was reported between 2–4 and 17–19 years [37], but with no growth models.

After reviewing the existing information on developmental pathways of spinal ossification centers [2, 3, 5, 6, 23, 40], we managed to find detailed morphometric data concerning only the C4 and L3 vertebrae [4, 33]. Thus, the present study is the first to provide completely novel reference values and growth dynamics for length, width, CSA, and volume of the three ossification centers of T6 vertebra in human fetuses of 115–265 mm CRL. As provided in both Tables 3, 4 and Fig. 4, the ossification center of the vertebral body offered a sharp contrast, being much larger than that of each neural process. As with the C4 and L3 vertebrae [4, 33], the growth dynamics for all the three ossification centers of T6 vertebra were all alike, because both their transverse and sagittal diameters increased logarithmically, while both their CSAs and volumes followed linearly. It is noteworthy, that the sagittal-to-transverse diameter ratio of the T6 body ossification center increased with gestational age from 0.81 ± 0.07 to 0.85 ± 0.08. It should also be emphasized that the T6 vertebral body and its ossification center grew in volume according to the four-degree polynomial (*y* = 19.158 + 0.0002 × age^4^ ± 7.942) and linear (*y* = −22.120 + 1.663 × age ± 1.869) functions, respectively. As a consequence, the relative size of the T6 body ossification center gradually decreased with age, from 0.28 ± 0.07 at 17 weeks to 0.21 ± 0.05 at 30 weeks of gestation.

According to Bareggi et al. [3], ossification centers of vertebral bodies were characterized by a faster ossification sequence than those of neural processes. As far as the neural processes are concerned, their left and right ossification centers developed symmetrically, with no laterality differences. On the right and left sides, both their lengths (*y* = −15.188 + 6.332 × ln(age) ± 0.629, *y* = −15.991 + 6.600 × ln(age) ± 0.629) and widths (*y* = −6.716 + 2.814 × ln(age) ± 0.362, *y* = −7.058 + 2.976 × ln(age) ± 0.323) grew in a natural logarithmic fashion. On the other hand, both their CSAs (*y* = −5.665 + 0.591 × age ± 1.251, *y* = −11.281 + 0.853 × age ± 1.653) and volumes (*y* = −9.279 + 0.849 × age ± 2.302, *y* = −16.117 + 1.155 × age ± 1.832) followed linearly. Ossification progression within the neural processes is relevant in the diagnosis of neural tube defects [5, 20, 49].

In accordance with age-specific reference values for T6 vertebra, such spinal abnormalities as hemivertebra, butterfly vertebra, block vertebrae, and spina bifida may ultrasonographically be diagnosed and monitored in utero [42]. Hemivertebra refers to a laterally based wedge-shaped vertebra with unilateral aplasia of one of the two chondrification centers within the vertebral body, resulting in substantial deformity [15, 21] of the spine in its sagittal and coronal alignment. Butterfly vertebra results from the failure of fusion of two chondrification centers that normally form one ossification center, with the persistent notochord separating them [10, 24]. Both hemivertebra and butterfly vertebra may be associated with skeletal anomalies [13], diastematomyelia [18], cardiac, urogenital and gastrointestinal tract anomalies [48], and some conditions including Jarcho–Levin, Klippel–Feil, VATER, VACTERL, and OEIS syndromes [39]. Block vertebrae are the consequence of their mal-segmentation and fusion through neighboring intervertebral discs. Spina bifida is characterized by a midline cleft between two neural processes. Furthermore, accurate knowledge on the normal growth of spinal ossification centers in fetuses may be useful in the prenatal detection of skeletodysplasias. This could potentially result in both delayed ossification centers and widespread demineralization, which are typical of osteogenesis imperfecta type II [38], achondrogenesis [36], and thanatophoric dysplasia type I [38]. Because of mutations in the gene for the tissue-nonspecific isozyme of alkaline phosphatase, in infants with life-threatening hypophosphatasia, inorganic pyrophosphate is accumulated extracellularly, consequently leading to rickets, osteomalacia, and finally to progressive chest and spine deformity [47]. On the other hand, it should be noticed that some 1–3 % of otherwise healthy children in the at-risk population of those aged 10–16 years, with a higher incidence of females, are affected by adolescent idiopathic scoliosis (AIS) [43, 44]. AIS is a complicated three-dimensional spinal deformity involving a structural, lateral, rotated curvature of the spine with the Cobb angle of at least 10°, vertebral body rotation and angulation of the ribs. In the posterior–anterior chest radiograms, the Cobb angle is between intersecting lines drawn vertical to the top of the uppermost affected vertebra and the bottom of the lowermost affected vertebra [43]. There is a close relationship between an increase in spinal curvature and the pubertal growth spurt [12, 43–45]. In AIS patients, longitudinal growth of vertebral bodies is disproportionate and much faster than in both sex-matched and age-matched controls. According to Weinstein et al. [44], progressive AIS may be due to spinal growth asymmetry, because of relative anterior spinal overgrowth attributed to endochondral ossification during the adolescent growth spurt. As reported by DiMeglio et al. [12], the pubertal diagram is characterized by two phases: a phase of acceleration (the first 2 years) and a phase of deceleration (the last 3 years). Therefore, peak growth velocity is the most critical period for AIS. Of note, most authors agree that curves with a thoracic apex are characterized by the highest prevalence of progression, ranging 58–100 % [43–45]. Furthermore, compared with female AIS patients, male patients revealed a lower tendency towards curve progression [41].

In summary, this is a cross-sectional study that documents the growth of T6 vertebra, including its three ossification centers in human fetuses of 115–265 mm CRL. Our reference values for the growing T6 vertebra may facilitate the diagnosis of many spinal disorders in human fetuses.

## Conclusions

1. Neither sex nor laterality differences are found in the morphometric parameters of evolving T6 vertebra and its three ossification centers.
2. The growth dynamics of the T6 vertebral body follow logarithmically for its height, and both sagittal and transverse diameters, linearly for its CSA, and four-degree polynomially for its volume.
3. The three ossification centers of T6 vertebra increase logarithmically in both transverse and sagittal diameters, and linearly in both CSA and volume.
4. The age-specific reference intervals for evolving T6 vertebra present the normative values of potential relevance in the diagnosis of congenital spinal defects.

## References

[1] Abe S, Suzuki M, Cho KH, Murakami G, Cho BH, Ide Y (2011). CD34-positive developing vessels and other structures in human fetuses: an immunohistochemical study. Surg Radiol Anat.

[2] Bagnall KM, Harris PF, Jones PRM (1979). A radiographic study of the human fetal spine 3. Longitudinal development of the ossification centers. J Anat.

[3] Bareggi R, Grill V, Zweyer M, Narducci P, Forabosco A (1994). A quantitative study on the spatial and temporal ossification patterns of vertebral centra and neural arches and their relationship to the fetal age. Ann Anat.

[4] Baumgart M, Szpinda M, Szpinda A (2012). New anatomical data on the growing C4 vertebra and its three ossification centers in human fetuses. Surg Radiol Anat.

[5] de Biasio P, Ginocchio G, Aicardi G, Ravera G, Venturini PL (2003). Ossification timing of sacral vertebrae by ultrasound in the mid-second trimester of pregnancy. Prenat Diagn.

[6] Budorick NE, Pretorius DH, Grafe MR, Lou KV (1991). Ossification of the fetal spine. Radiology.

[7] Chen Y, Zhuang Z, Qi W, Yang H, Chen Z, Wang X, Kong K (2011). A three-dimensional study of the atlantodental interval in a normal Chinese population using reformatted computed tomography. Surg Radiol Anat.

[8] Cho KH, Rodríguez-Vázquez JF, Kim JH, Abe H, Murakami G, Cho BH (2011). Early fetal development of the human cerebellum. Surg Radiol Anat.

[9] Choufani E, Jouve JL, Pomero V, Adalian P, Chaumoitre K, Panuel M (2009). Lumbosacral lordosis in fetal spine: genetic or mechanic parameter. Eur Spine J.

[10] Chrzan R, Podsiadlo L, Herman-Sucharska I, Urbanik A, Bryll A (2010). Persistent notochordal canal imitating compression fracture—plain film, CT and MR appearance. Med Sci Monit.

[11] Cui G, Watanabe K, Hosogane N, Tsuji T, Ishii K, Nakamura M, Toyama Y, Chiba K, Lenke LG, Matsumoto M (2012). Morphologic evaluation of the thoracic vertebrae for safe free-hand pedicle screw placement in adolescent idiopathic scoliosis: a CT-based anatomical study. Surg Radiol Anat.

[12] DiMeglio A, Canavese F, Charles YP (2011). Growth and adolescent idiopathic scoliosis: when and how much. J Pediatr Orthop.

[13] Goldstein I, Makhoul IR, Weissman A, Drugan A (2005). Hemivertebra: prenatal diagnosis, incidence and characteristics. Fetal Diagn Ther.

[14] Iffy L, Jakobovits A, Westlake W, Wingate MB, Caterini H, Kanofsky P, Menduke H (1975). Early intrauterine development: I. The rate of growth of Caucasian embryos and fetuses between the 6th and 20th weeks of gestation. Pediatrics.

[15] Jalanko T, Rintala R, Puisto V, Helenius I (2011). Hemivertebra resection for congenital scoliosis in young children: comparison of clinical, radiographic, and health-related quality of life outcomes between the anteroposterior and posterolateral approaches. Spine.

[16] Jin ZW, Song KJ, Lee NH, Nakamura T, Fujimiya M, Murakami G, Cho BH (2011). Contribution of the anterior longitudinal ligament to ossification and growth of the vertebral body: an immunohistochemical study using the human fetal lumbar vertebrae. Surg Radiol Anat.

[17] Kaplan KM, Spivak JM, Bendo JA (2005). Embryology of the spine and associated congenital abnormalities. Spine J.

[18] Leug YL, Buton N (2005). Combined diastematomyelia and hemivertebra. A review of the management at a single centre. J Bone Jt Surg.

[19] Margolis AJ, Voss RG (1968). A method for radiologic detection of fetal maturity. Am J Obstet Gynecol.

[20] Masharawi Y, Salame K (2011). Shape variation of the neural arch in the thoracic and lumbar spine: characterization and relationship with the vertebral body shape. Clin Anat.

[21] Masharawi Y, Salame K, Mirovsky Y, Peleg S, Dar G, Steinberg N, Hershkovitz I (2008). Vertebral body shape variation in the thoracic and lumbar spine: characterization of its asymmetry and wedging. Clin Anat.

[22] Matsumoto M, Okada E, Kaneko Y, Ichihara D, Watanabe K, Chiba K, Toyama Y, Fujiwara H, Momoshima S, Nishiwaki Y, Hashimoto T, Takahata T (2011). Wedging of vertebral bodies at the thoracolumbar junction in asymptomatic healthy subjects on magnetic resonance imaging. Surg Radiol Anat.

[23] Noback CR, Robertson GG (1951). Sequences of appearance of ossification centers in the human skeleton during the first five prenatal months. Am J Anat.

[24] Patinharayil G, Han CW, Marthya A, Meethall KC, Surendran S, Rudrappa GH (2008). Butterfly vertebra: an uncommon congenital spinal anomaly. Spine.

[25] Saraga-Babić M (1991). Development of the notochord in normal and malformed human embryos and fetuses. Int J Dev Biol.

[26] Schild RL, Wallny T, Fimmers R, Hansmann M (1999). Fetal lumbar spine volumetry by three-dimensional ultrasound. Ultrasound Obstet Gynecol.

[27] Schild RL, Wallny T, Fimmers R, Hansmann M (2000). The size of the fetal thoracolumbar spine: a three-dimensional ultrasound study. Ultrasound Obstet Gynecol.

[28] Skawina A, Litwin JA, Gorczyca J, Miodoński AJ (1997). The architecture of internal blood vessels in human fetal vertebral bodies. J Anat.

[29] Standring S (2008). Gray’s anatomy. The anatomical basis of clinical practice.

[30] Szpinda M, Baumgart M, Szpinda A (2013) Cross-sectional study of the C1–S5 vertebral bodies in human fetuses. Arch Med Sci (in press) 10.5114/aoms.2013.37086 PMC4379359 25861306

[31] Szpinda M, Baumgart M, Szpinda A, Woźniak A, Małkowski B, Wiśniewski M, Mila-Kierzenkowska C, Króliczewski D (2012). Cross-sectional study of the ossification center of the C1–S5 vertebral bodies. Surg Radiol Anat.

[32] Szpinda M, Baumgart M, Szpinda A, Woźniak A, Mila-Kierzenkowska C (2013) Cross-sectional study of the neural ossification centers of vertebrae C1–S5 in the human fetus. Surg Radiol Anat. doi: 10.1007/s00276-013-1093-5 10.1007/s00276-013-1093-5 PMC3784062 23455365

[33] Szpinda M, Baumgart M, Szpinda A, Woźniak A, Mila-Kierzenkowska C (2013) New patterns of the growing L3 vertebra and its 3 ossification centers in human fetuses—a CT, digital and statistical study. Med Sci Monit (in press) 10.12659/MSMBR.883956 PMC3692385 23778313

[34] Szpinda M, Daroszewski M, Woźniak A, Szpinda A, Mila-Kierzenkowska C (2012). Tracheal dimensions in human fetuses: an anatomical, digital and statistical study. Surg Radiol Anat.

[35] Szpinda M, Szpinda A, Woźniak A, Daroszewski M, Mila-Kierzenkowska C (2012). The normal growth of the common iliac arteries in human fetuses—an anatomical, digital and statistical study. Med Sci Monit.

[36] Taner MZ, Kurdoglu M, Taskiran C, Onan MA, Gunaydin G, Himmetoglu O (2008). Prenatal diagnosis of achondrogenesis type I: a case report. Cases J.

[37] Tulsi RS (1971). Growth of the human vertebral column: an osteological study. Acta Anat.

[38] Ulla M, Aiello H, Cobos MP, Orioli I, García-Mónaco R, Etchegaray A, Igarzábal ML, Otaño L (2011). Prenatal diagnosis of skeletal dysplasias: contribution of three-dimensional computed tomography. Fetal Diagn Ther.

[39] Varras M, Akrivis C (2010). Prenatal diagnosis of fetal hemivertebra at 20 weeks’ gestation with literature review. Int J Gen Med.

[40] Vignolo M, Ginocchio G, Parodi A, Torrisi C, Pistorio A, Venturini PL, Aicardi G, de Biasio P (2005). Fetal spine ossification: the gender and individual differences illustrated by ultrasonography. Ultrasound Med Biol.

[41] Wang WJ, Sun X, Wang ZW, Qiu XS, Liu Z, Qiu Y (2012). Abnormal anthropometric measurements and growth pattern in male adolescent idiopathic scoliosis. Eur Spine J.

[42] Wax JR, Watson WJ, Miller RC, Ingardia CJ, Pinette MG, Cartin A, Grimes CK, Blackstone J (2008). Prenatal sonographic diagnosis of hemivertebrae: associations and outcomes. J Ultrasound Med.

[43] Weinstein SL (1994). The pediatric spine: principles and practice.

[44] Weinstein SL, Dolan LA, Cheng JCY, Danielsson A, Morcuende JA (2008). Adolescent idiopathic scoliosis. Lancet.

[45] Weinstein SL, Dolan SA, Spratt KF, Peterson KK, Spoonamore MJ, Ponseti IV (2003). Health and function of patients with untreated idiopathic scoliosis: a 50-year natural history study. JAMA.

[46] Weisz B, Achiron R, Schindler A, Eisenberg VH, Lipitz S, Zalel Y (2004). Prenatal sonographic diagnosis of hemivertebra. J Ultrasound Med.

[47] Whyte MP, Greenberg CR, Salman NJ, Bober MB, McAlister WH, Wenkert D, Van Sickle BJ, Simmons JH, Edgar TS, Bauer ML, Hamdan MA, Bishop N, Lutz RE, McGinn M, Craig S, Moore JN, Taylor JW, Cleveland RH, Chanley WR, Lim R, Thacher TD, Mayhew JE, Downs M, Millan JL, Skrinar AM, Crine P, Landy H (2012). Enzyme-replacement therapy in life-threatening hypophosphatasia. N Engl J Med.

[48] Zelop CM, Pretorius DH, Benacerraf BR (1993). Fetal hemivertebrae: associated anomalies, significance, and outcome. Obstet Gynecol.

[49] Zhuang Z, Xie Z, Ding S, Chen Y, Luo J, Wang X, Kong K (2012). Evaluation of thoracic pedicle morphometry in a Chinese population using 3D reformatted CT. Clin Anat.

